# Cu‐Co Dual Sites Tandem Synergistic Effect Boosting Neutral Low Concentration Nitrate Electroreduction to Ammonia

**DOI:** 10.1002/advs.202416386

**Published:** 2025-02-17

**Authors:** Wenhao Yang, Ziwei Chang, Xu Yu, Ping Wu, Ruxiang Shen, Lianzhou Wang, Xiangzhi Cui, Jianlin Shi

**Affiliations:** ^1^ State Key Laboratory of High‐Performance Ceramics and Superfine Microstructure Shanghai Institute of Ceramics Chinese Academy of Sciences Shanghai 200050 P. R. China; ^2^ Center of Materials Science and Optoelectronics Engineering University of Chinese Academy of Sciences Beijing 100049 P. R. China; ^3^ School of Physical Science and Technology Shanghai Tech University Shanghai 201210 P. R. China; ^4^ Nanomaterials Centre School of Chemical Engineering and Australian Institute for Bioengineering and Nanotechnology The University of Queensland St Lucia Brisbane QLD 4072 Australia; ^5^ School of Chemistry and Materials Science Hangzhou Institute for Advanced Study University of Chinese Academy of Sciences Hangzhou 310024 P. R. China

**Keywords:** active hydrogen, dual sites, electrochemical nitrate reduction, neutral low concentration nitrate, tandem catalysis

## Abstract

Electrochemical nitrate reduction reaction (NO_3_^−^RR) has emerged as an alternative strategy for wastewater treatment and ammonia production in neutral low‐concentration nitrate. However, the electrocatalyst faces the challenge of limited NO_3_^−^ distribution and deficient active hydrogen (H_ads_) on the catalyst surface resulting from the low concentration of NO_3_^−^ and the difficulty of water splitting under neutral conditions. Here, a Cu‐Co dual sites tandem synergistic catalysis mechanism has been proposed by doping Cu into CoP to facilitate the adsorption and conversion of NO_3_^−^ on Cu and to accelerate the water splitting on CoP leading to the significantly high NO_3_^−^RR performance. The designed Cu‐CoP catalyst exhibits an ammonia yield of 7.65 mg h^−1^ cm^−2^ and a Faraday efficiency of 85.1% at −1.0 V under neutral low‐concentration nitrate (10 m m), which is the highest ammonia yield in the reported data. In situ characterization and theoretical calculations confirm the tandem synergistic effect, in which the Cu site favors the adsorption and activation of NO_3_^−^ to form NO_2_^−^, and concurrently modulates the electronic structure of the Co site with optimized H_ads_ adsorption resulting in the significantly enhanced NO_3_^−^RR at neutral low concentration nitrate.

## Graphical abstract

A novel Cu‐Co dual sites tandem synergistic catalysis mechanism is proposed to facilitate the adsorption and activation of NO_3_^‐^ to form NO_2_^‐^ on Cu and to efficiently generate H_ads_ for reaction with NO_2_^‐^ by accelerating water splitting, leading to the significantly improved performance of the Cu‐CoP in the electroreduction of neutral low concentrations of NO_3_^‐^ to NH_3_.

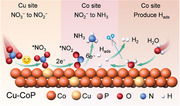

## 1 Introduction

Ammonia (NH_3_) as one of the largest chemicals produced in the world, is expected to be the next generation of hydrogen‐rich fuel owing to its high energy density and carbon‐free emission.^[^ ^1^ ^]^ The current production of NH_3_ is mainly via the Haber–Bosch method under high temperature and pressure conditions, which leads to more than 2% of the world's energy consumption and over 500 million tons of carbon dioxide emissions per year.^[^ ^2^ ^]^ Meanwhile, industrial wastewater discharges and the misuse of fertilizers result in the widespread distribution of nitrate (NO_3_^−^) pollutants in surface waters and underground aquifers, posing a serious threat to human health.^[^ ^3^ ^]^ Therefore, the conversion of NO_3_^−^ to high‐value‐added NH_3_ by electrocatalysis method at ambient temperature and pressure provides both environmental remediation and energy economy advantages. The NO_3_^−^RR for NH_3_ production has made great progress in basic research, but most of the reported catalysts are operated in high NO_3_^−^ concentration (≥100 mm) conditions, which require additional consideration of the expensive cost of concentrating the NO_3_^−^ solution.^[^ ^4^ ^]^ The majority of NO_3_^−^ sources, such as semiconductor industry wastewater (<13 mM), textile industry wastewater (<8 mm), and contaminated groundwater (<2 m m), have low NO_3_^−^ concentrations.^[^ ^5^ ^]^ Thus the electroreduction of neutral low concentrations of NO_3_^−^ to NH_3_ is practical and necessary, which faces extreme challenges due to the limited distribution of NO_3_^−^ on the catalyst surface and the presence of competitive hydrogen evolution reaction (HER).

To address the difficulty of NO_3_^−^ adsorption on the catalyst surface in low concentration NO_3_^−^RR, recent studies have proposed strategies involving pulsed potentials,^[^ ^6^ ^]^ NO_3_^−^ enrichment,^[^ ^7^ ^]^ and built‐in electric fields^[^ ^8^ ^]^ to increase the localized NO_3_^−^ concentration on the catalyst surface, which improves the NH_3_ yield and Faraday efficiency (FE). Comparably, the strategy to improve the intrinsic activity of the catalyst by its design may be simpler and more effective. Meanwhile, NO_3_^−^RR involves a series of nitrogen‐oxygen intermediates being attacked by H_ads_ produced via water splitting for deoxygenation and hydrogenation to produce NH_3,_^[^ ^9^ ^]^ and the pH of low concentration NO_3_^−^ wastewater often tends to be neutral, which requires a more negative potential to generate H_ads_ via the Volmer step (H_2_O + e^−^ →H_ads_ + OH^−^) resulting in the H_ads_ tend to dimerize to produce hydrogen.^[^ ^10^ ^]^ Therefore, the development of a catalyst with strong adsorption capacity for NO_3_^−^, promoting water splitting and effectively inhibiting H‐H dimerization is crucial for NH_3_ production from NO_3_^−^RR at neutral low concentrations.

**Scheme 1:**
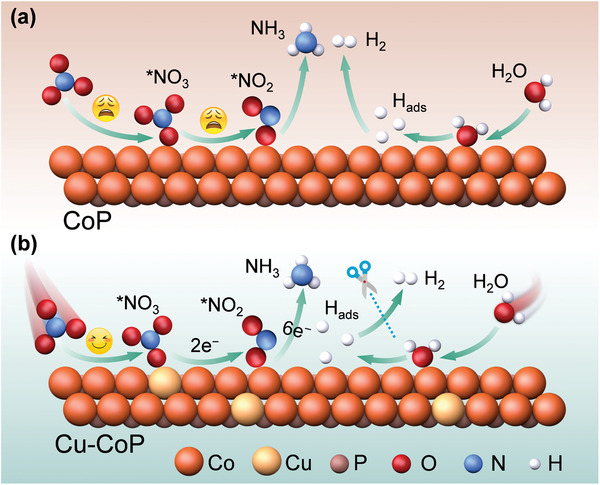
Schematic illustration of the NO_3_^−^RR process over the CoP a) and Cu‐CoP b) catalyst systems.

The transition metal phosphides with high electrical conductivity and platinum‐like electronic structure enable efficient dissociation of water to produce H_ads_ to participate in the NO_3_^−^RR process.^[^ ^11^ ^]^ Theoretical calculations demonstrate that Co atoms have the lowest dissociation energy barrier for nitrogen‐oxygen intermediates (^*^NO_2_, ^*^NO) of all metal species, resulting in a high selectivity for NH_3_.^[^ ^12^ ^]^ Meanwhile, some recent studies have verified the activity of CoP toward NO_3_^−^RR.^[^ ^13^ ^]^ Unfortunately, however, the relatively weak adsorption capacity for NO_3_^−^ at CoP makes it difficult to convert low concentration of NO_3_^−^ to nitrite (NO_2_^−^),^[^ ^14^ ^]^ resulting in HER (**Scheme** **1**a). Excellent energy level matching between the highly occupied d‐orbitals of Cu and the lowest unoccupied molecular π^*^ orbital (LUMO π^*^) of NO_3_^−^ facilitates the adsorption and reduction of NO_3_^−^ to NO_2_^−^,^[^ ^15^ ^]^ and Cu can be introduced into the CoP to provide an adequate supply of NO_2_^−^ for the subsequent deoxygenation and hydrogenation processes. Based on the basic electrochemical property analysis of Cu and Co, the performance of Cu‐doped CoP toward NO_3_^−^RR at neutral low concentrations is expected to be significantly improved, which has not been reported as far as we know.

In this work, we proposed a Cu‐Co dual sites tandem synergistic catalysis mechanism by doping Cu into CoP to facilitate the adsorption and conversion of NO_3_^−^ on Cu and to optimize the adsorption of H_ads_ on CoP leading to the significantly high NO_3_^−^RR performance (Scheme 1b). Thus, the designed Cu‐CoP catalyst demonstrates an NH_3_ yield of 7.65 mg h^−1^ cm^−2^ and FE of 85.1% at −1.0 V vs RHE at neutral low concentration of nitrate (10 mM), which is the highest NO_3_^−^RR performance reported so far at the same conditions. In situ characterization and theoretical calculations confirmed the tandem synergistic effect between Cu and Co dual sites, in which the strong adsorption of NO_3_^−^ on the Cu site results in preferential NO_3_^−^ to NO_2_^−^ conversion and the ability to desorb NO_2_^−^ more readily, and the desorbed NO_2_^−^ was re‐adsorbed on the Co site to be converted to NH_3_. Concurrently, the modulating effect of Cu on the electronic structure of Co was beneficial in facilitating the water splitting to optimize the adsorption of H_ads_ preventing H_2_ release thus leading to high NO_3_^−^RR electrocatalytic activity.

## 2 Results and Discussion

### 2.1 Preparation and Characterization

**Figure 1:**
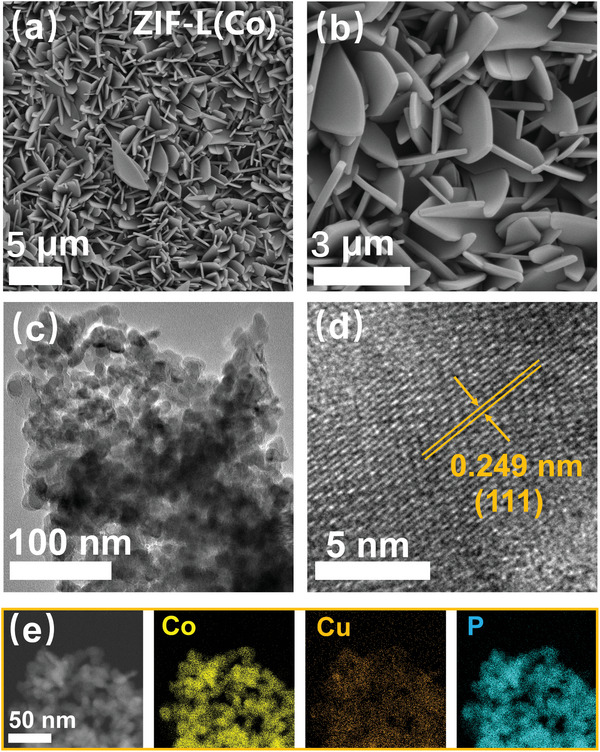
SEM images of ZIF‐L(Co) a, b) at different magnifications; TEM c) and HRTEM d) images of Cu‐CoP; e) HAADF and corresponding EDS elemental mapping images of Cu‐CoP.

To avoid the use of polymer binders and to expose more active sites, ZIF‐L(Co) precursors were prepared on nickel foam substrates using a simple solution method,^[^ ^16^ ^]^ and followed by a subsequent electrochemically‐driven cation‐exchange (ED‐CE) strategy^[^ ^17^ ^]^ and in‐situ phosphating process to prepare Cu‐CoP. The scanning electron microscopy (SEM) images show that the ZIF‐L(Co) were arranged in the form of leaves on the surface of nickel foam (**Figure** **1**a,b), and after ion exchange as well as phosphating process, the surface of the nano‐leaves becomes rough and porous (Figures S1, and S2, Supporting Information), which is conducive to increase the electrochemically active area, further transmission electron microscopy (TEM) images show that the surface of the nano‐leaves was covered with many tiny nanosheets (Figure 1c).^[^ ^18^ ^]^ High‐resolution TEM (HRTEM) images show that the interplanar spacing of Cu‐CoP is 0.249 nm (Figure 1d), which corresponds to the (111) plane of CoP, and it is larger compared to the standard CoP (111) plane spacing (0.247nm), which may be attributed to the introduction of Cu that expands the CoP lattice.^[^ ^19^ ^]^ The elemental mapping in Figure 1e shows a uniform distribution of Cu, Co, and P elements in Cu‐CoP.

**Figure 2:**
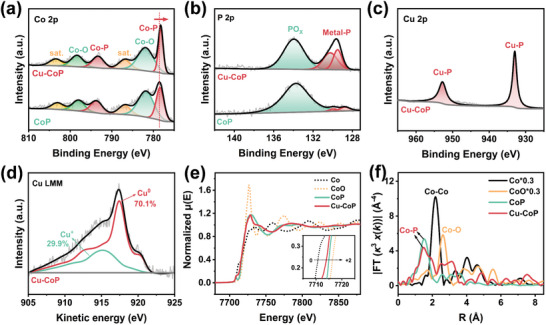
High‐resolution XPS spectra of a) Co 2p, b) P 2p for Cu‐CoP and CoP; high‐resolution c) Cu 2p XPS and d) Cu LMM AES spectra of Cu‐CoP; e) Co K‐edge XANES spectra and the corresponding f) EXAFS results of Co K‐edge for Cu‐CoP and the reference compounds.

The X‐ray diffraction (XRD) pattern of CoP and Cu‐CoP electrode is shown in Figure S3 (Supporting Information), and the peaks at 44.6°, 51.9°, and 76.4° are attributed to the basal nickel foam, the peaks at 40.8°, 47.3°, 54.2° are attributed to the nickel phosphide formed during the phosphating process of the nickel foam. The diffraction peaks of CoP at 36.1°, 39.2°, 46.9° (covered by the peaks of Ni_2_P), and 49.1° can be attributed to the (111), (201), (112), and (202) crystal planes, respectively. While those of the corresponding crystal planes of Cu‐CoP are positioned at 36°, 39.1°, 46.2°, and 48.5°, which shift negatively with respect to CoP, indicating that Cu‐CoP has a larger lattice spacing, agreeing well with the HRTEM results.^[^ ^20^ ^]^ X‐ray photoelectron spectroscopy (XPS) was used to investigate the surface composition and binding structure of the catalysts. The XPS spectra of Co and P elements in Cu‐CoP and CoP show almost identical peak positions (**Figure** **2**a,b). The high‐resolution Co 2p XPS spectra of Cu‐CoP show characteristic peaks at binding energies of 778.3 and 793.3 eV respectively attributed at Co 2p_3/2_ and Co 2p_1/2_ of the Co─P bond, and the characteristic peaks with binding energies of 782.0 and 798.4 eV are attributed to Co‐PO_x_ bonds (metal phosphates formed by exposure to air) of Co 2p_3/2_ and Co 2p_1/2_, respectively.^[^ ^13a^ ^]^ Moreover, the Co 2p spectra of Cu‐CoP are slightly negatively shifted compared to CoP, indicating electron transfer from Cu to Co due to the introduction of the Cu element. The high‐resolution P 2p spectra show that the characteristic peaks of the binding energies at 129.5 and 130.3 eV belong to Metal‐P bonds, and the characteristic peak at 133.9 eV belongs to Metal‐PO_x_ bonds.^[^ ^21^ ^]^ The high‐resolution Cu 2p XPS spectra (Figure 2c) and Cu LMM Auger spectra (Figure 2d) show that the peaks at 933.4 and 952.8 eV can be attributed to the formation of Cu─P bonds by the doped Cu species, and the Cu species are primarily Cu^0^ (70.1%) and minor amounts of Cu^+^ (29.9%).^[^ ^22^ ^]^

The normalized X‐ray absorption near‐edge structure (XANES) and the Fourier‐transform extended X‐ray absorption fine structure (EXAFS) spectra of the Co K‐edge were obtained to explore the local electronic or atomic structure of Cu‐CoP. In Figure 2e, the absorption edge energies of the Co K‐edge in both CoP and Cu‐CoP are intermediate between those of standard Co and CoO, indicating that the valence state of the element Co is between 0 and +2 valence. Moreover, the valence state of Co is lower in Cu‐CoP compared to CoP,^[^ ^16^ ^]^ and these analyses are in agreement with the XPS results, indicating that the introduction of Cu induced the electron redistribution on Co, which effectively regulated the electronic structure of Co, thus facilitating the deoxygenation and hydrogenation of the intermediates in the NO_3_^−^RR process. In Figure 2f, the Co‐Co coordination (2.18 Å) is not observed in Cu‐CoP,^[^ ^23^ ^]^ indicating that the Co element exists as a mononuclear metal center in Cu‐CoP. Meanwhile, Cu‐CoP shows a similar radial distribution function as that of CoP, with a Co─P bond at 1.5 Å.

### 2.2 Electrocatalytic NO_3_^−^RR Performance

The NO_3_^−^RR performance of the obtained catalysts was evaluated using a three‐electrode system. All potentials are referenced to a reversible hydrogen electrode (RHE). To simulate the actual semiconductor industry wastewater and textile wastewater, the nitrate test concentration was set at 10 mm. Modulation of Cu doping in Cu‐CoP catalysts by controlling the charge of the ED‐CE process. As shown in Figure S4 (Supporting Information), Cu‐CoP catalysts with different Cu doping were synthesized under the experimental conditions with total charges of 0, 2, 4, 6, and 8 C cm^−2^ and NH_3_ yields and FE were measured at a potential of −0.8 V, respectively. The NH_3_ yields and FE show a volcanic profile trend with increasing total charge, where the highest NH_3_ yields and FE are observed for 4Cu‐CoP. All of the Cu‐CoP in the latter article have a charge of 4C during the ED‐CE process.

**Figure 3:**
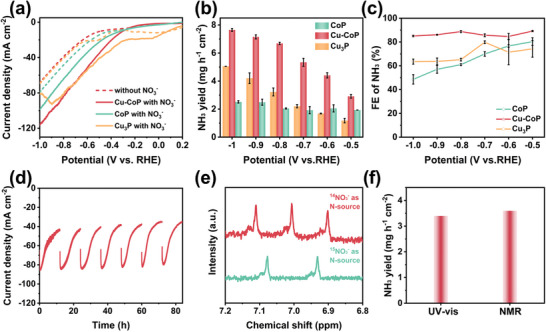
a) LSV curves for the Cu‐CoP, CoP, and Cu_3_P recorded at a scan rate of 50 mV s^−1^ in 0.1 m K_2_SO_4_ with and without 0.01 m KNO_3_; NH_3_ yield b) and FE c) at different applied potentials in 0.1 m K_2_SO_4_ + 0.01 m KNO_3_; d) Long‐term NO_3_^−^RR electrolysis over Cu‐CoP catalyst at −0.8 V (vs. RHE) for 84 h; e) ^1^H NMR spectra of the electrolyte after testing using ^15^NO_3_^−^ and ^14^NO_3_^−^ as nitrogen sources; f) NH_3_ yield of NO_3_^−^RR at −0.6 V (vs. RHE) using ^14^NO_3_^−^ as the nitrogen source, detected by salicylic acid method and ^1^H NMR spectroscopy.

Linear scanning voltammetry (LSV) was used to initially determine the NO_3_^−^ reduction activity of the samples. In **Figure** **3**a, the current densities of Cu‐CoP, CoP, and Cu_3_P in 0.01 M NO_3_^−^ electrolyte are all higher than that in the absence of added NO_3_^−^, implying that all three catalysts are catalytically active for NO_3_^−^ reduction. It is interesting to note that the current of the Cu_3_P catalyst becomes sharply smaller at −0.9 V. This is due to the poisoning of Cu_3_P by NO_2_^−^, which leads to a sharp decrease in its catalytic performance.^[^ ^24^ ^]^ The product distribution of NO_3_^−^RR over the three catalysts in the potential range of −0.5 to −1.0 V was investigated by UV colorimetry (Figures S5–S7, Supporting Information). As shown in Figure 3b, the NH_3_ yields of Cu‐CoP and CoP gradually increased with the negative shift of the applied potential, while that of Cu_3_P changed inconspicuously, indicating that the Co site produces plenty of reactive hydrogen with the applied potential, and NO_3_^−^RR of NH_3_ production via the H_ads_ mediated pathway.^[^ ^9^ ^]^ Meanwhile, Cu‐CoP showed a higher NH_3_ yield than CoP and Cu_3_P, reaching a maximum of 7.65 mg h^−1^ cm^−2^ at −1.0 V, which is the best performance among similar electrolytes reported currently (Tabel S1, Supporting Information).^[^ ^4^, ^14^, ^25^ ^]^ Figure 3c shows that the FE of Cu‐CoP is comprehensively improved over the entire range of applied potentials compared to CoP, producing ammonia at a maximum FE of 89.2% at −0.5 V. Interestingly, the FE of CoP decreases sharply with increasing applied potential, which may be attributed to its insufficient utilization of H_ads_ in the NO_3_^−^RR process, leading to the production of large amounts of H_2_ by‐products from H‐H dimerization. The electrochemical impedance spectra and electrochemically active surface area tests showed that Cu‐CoP shares similar charge transfer resistance and electrochemically active surface area with CoP (Figures S8, and S9, Supporting Information), which indicates that the efficient performance of Cu‐CoP for NH_3_ production derives from its high intrinsic activity. Furthermore, Cu‐CoP exhibits a long‐term stability of more than 80h (Figure 3d). The current density decay is attributed to NO_3_^−^ depletion during continuous electrolysis, which recovers when the electrolyte is replaced.^[^ ^26^ ^]^ Furthermore, when we connected 3 L of electrolyte outside the electrolytic cell via a peristaltic pump to provide circulating electrolyte to minimize the effect of NO_3_^−^ consuming on the current density, the current density of the Cu‐CoP catalyst showed no significant degradation in 9 h (Figure S10, Supporting Information), indicating its excellent stability toward NO_3_^−^RR. TEM and XPS characterization of the material after stability measurements showed that the morphology, lattice spacing, and binding energy of Cu‐CoP were essentially the same as before electrochemical testing (Figure S11, Supporting Information). These results indicate that Cu‐CoP has high durability to NO_3_^−^RR. Moreover, the N‐NO_3_^−^ concentration was reduced to 1.74 mg L^−1^ in 220 min by using a Cu‐CoP catalyst (Figure S12, Supporting Information), which is lower than 3 mg L^−1^ under the Safe Drinking Water Act of the US Environmental Protection Agency, indicating the application potential of Cu‐CoP toward NO_3_^−^RR.

The cleaned nickel foam was phosphate using the same phosphating conditions, and the NH_3_ yield was 0.215 mg h^−1^ cm^−2^, which was far lower than that of Cu‐CoP, indicating the minor contribution of the phosphate nickel foam substrate to the NH_3_ yield (Figure S13, Supporting Information). To ensure that NH_3_ was produced by NO_3_^−^RR and not from environmental pollution, a series of controlled experiments were conducted. Cu‐CoP electrode did not detect ammonia in electrolytes containing 0.01 m KNO_3_ at open‐circuit potential and in electrolytes without KNO_3_ at −0.8 V. These results indicate that trace nitrogenous impurities in the electrolyte and self‐electrolysis of the catalyst do not confuse the NH_3_ detection in the experiments. The isotopic labeling combined with nuclear magnetic resonance (NMR) was used to further verify that the ammonia originated from NO_3_^−^ (Figure 3e; Figure S14, Supporting Information). With ^15^NO_3_^−^ as the feed reactant, the ^1^H NMR spectrum showed a typical double peak of ^15^NH_4_^+^. The typical triple peak was presented when the feed was ^14^NO_3_^−^. Furthermore, the salicylic acid method was compared with NMR‐calculated NH_3_ yields with highly consistent results (Figure 3f).

### 2.3 Mechanism Study

**Figure 4:**
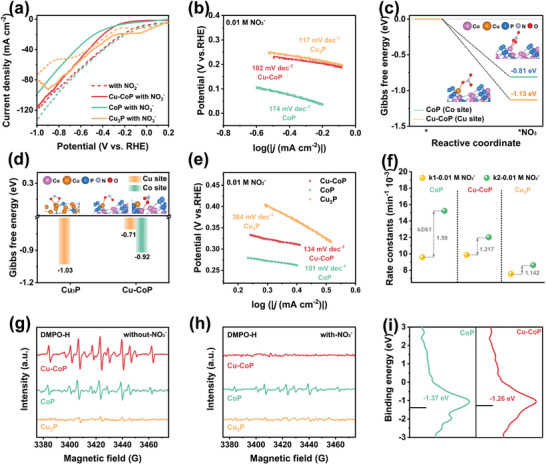
a) LSV curves for the Cu‐CoP, CoP, and Cu_3_P recorded at a scan rate of 50 mV s^−1^ in 0.1 m K_2_SO_4_ with 0.01 m KNO_3_ or 0.01 m KNO_2_; b) Tafel slopes of Cu‐CoP, CoP and Cu_3_P in 0.1 m K_2_SO_4_ with 0.01 m KNO_3_; c) Calculation of the adsorption energy of NO_3_^−^ molecules by Co sites of CoP and Cu sites of Cu‐CoP; d) Calculation of the adsorption energy of NO_2_^−^ molecules by Cu sites of Cu_3_P, as well as by Cu and Co sites of Cu‐CoP; e) Tafel slopes of Cu‐CoP, CoP and Cu_3_P in 0.1 m K_2_SO_4_ with 0.01 m KNO_2_; f) Rate constants for NO_3_^−^ and NO_2_^−^ consumption on Cu‐CoP, CoP, Cu_3_P catalysts (rate constants k are calculated based on the concentration consumption of NO_3_^−^ and NO_2_^−^ ions); ESR spectra of the catalyst with DMPO in 0.1 m K_2_SO_4_ g) and 0.1 m K_2_SO_4_ with 0.01 m KNO_3_ h) electrolyte at −0.8 V after 5 min NO_3_^−^RR in Ar atmosphere; i) Calculated d‐band center values of Cu‐CoP and CoP.

The reduction of NO_3_^−^ to NH_3_ can be considered as a two‐step reaction in tandem, the initial conversion of NO_3_^−^ to NO_2_^−^ and the subsequent conversion of NO_2_^−^ to NH_3_, thus it is crucial to promote the fast reaction of these two steps simultaneously. The prepared catalysts respectively performed LSV tests in NO_3_^−^ or NO_2_^−^ containing electrolytes to gain a preliminary understanding of the improvement of NO_3_^−^RR performance on Cu‐CoP.^[^ ^27^ ^]^ As shown in **Figure** **4**a, in the NO_2_^−^ containing electrolyte, the current densities of CoP and Cu‐CoP were higher than that of Cu_3_P, indicating that CoP and Cu‐CoP had a better activity for NO_2_^−^ electroreduction. It is worth noting that NO_2_^−^ can poison the Cu_3_P catalyst and sharply decrease its performance, and when the second LSV test was performed on Cu_3_P in the NO_2_^−^ containing electrolyte, the current density decreased dramatically compared to the first one, approaching the HER performance of Cu_3_P (Figure S15, Supporting Information). Whereas, in NO_3_^−^ containing electrolytes, CoP exhibited the lowest current density, indicating that a single Co site has limited NO_3_^−^RR activity.

The rate‐determining step (RDS) of NO_3_^−^RR for the three catalysts was analyzed by electrodynamics (Figure 4b). The Tafel slope of CoP was 174 mV dec^−1^, which was much higher than 120 mV dec^−1^, indicating that the NO_3_^−^RR process of CoP was limited by the initial adsorption and activation of NO_3_^−^, whereas the Tafel slopes of Cu‐CoP and Cu_3_P were 102 and 117 mV dec^−1^ (lower than 120 mV dec^−1^), respectively, which proved that Cu‐CoP and Cu_3_P had faster kinetics of NO_3_^−^ reduction.^[^ ^28^ ^]^ Furthermore, the positive effect of introducing Cu sites in CoP was verified by calculating the adsorption energies of NO_3_^−^ and NO_2_^−^ molecules on different sites. As shown in Figure 4c, the adsorption energy of NO_3_^−^ on CoP was −0.81 eV, whereas that on the introduced Cu site was −1.13 eV, indicating that the Cu site in Cu‐CoP has a stronger adsorption capacity of NO_3_^−^ and converts NO_3_^−^ to NO_2_^−^. The calculation of NO_2_^−^ adsorption energy shows that the weak adsorption of NO_2_^−^ at the Cu sites in Cu‐CoP facilitates its desorption into the electrolyte, which can effectively avoid the poisoning effect of NO_2_^−^ on Cu, and the strong adsorption of NO_2_^−^ at the Co site facilitates the capture of NO_2_^−^ produced on Cu for the subsequent hydrogenation process (Figure 4d).^[^ ^22^ ^]^ The electrokinetic analysis in the NO_2_^−^ containing electrolyte showed Tafel slopes of 134 and 101 mV dec^−1^ for Cu‐CoP and CoP, respectively, which were much lower than that of 384 mV dec^−1^ for Cu_3_P, indicating that the Co site has much faster kinetics for the reduction of NO_2_^−^ to NH_3_. (Figure 4e).^[^ ^29^ ^]^

The above results indicate that the Cu site of the Cu‐CoP catalyst mainly converts NO_3_^−^ to NO_2_^−^ and the Co site is the subsequent conversion of NO_2_^−^ to NH_3_. To verify this hypothesis, we further evaluated the rate constants k1 and k2 for the consumption of NO_3_^−^ and NO_2_^−^ by each catalyst, respectively (Figure 4f).^[^ ^14^ ^]^ The k1 reflects the rate of consumption of NO_3_^−^, which includes the conversion of NO_3_^−^ to NO_2_^−^ and NH_3_, whereas the k2 reflects the rate of consumption of NO_2_^−^, which is only the conversion of NO_2_^−^ to NH_3_. Interestingly, the k1/k2 ratio for Cu_3_P is 1.142, indicating that the NO_3_^−^RR rate on Cu_3_P is close to the NO_2_^−^RR rate. While in a chemical reaction, the reaction rate is usually determined by the slowest reaction step, it is the faster reaction rate NO_3_^−^ reduction to NO_2_^−^a step on Cu_3_P that leads to NO_2_^−^ reduction to NH_3_ being the RDS, and therefore exhibits reaction rates in NO_3_^−^RR close to NO_2_^−^RR. In both CoP and Cu‐CoP, the k2 values are much larger than those of Cu_3_P, indicating that the Co sites rapidly reduce NO_2_^−^ to NH_3_, and during performance tests, the CoP and Cu‐CoP both exhibit very low FE of the NO_2_^−^ product (Figure S16, Supporting Information). Cu‐CoP combines the positive properties of Cu_3_P and CoP in the conversion of NO_3_^−^ to NO_2_^−^ and NO_2_^−^ to NH_3_, respectively, and the synergistic tandem catalyzation of the two active sites makes Cu‐CoP exhibit the best NO_3_^−^RR performance.

The NO_3_^−^RR involves a series of nitrogen‐oxygen intermediates deoxygenated and hydrogenated to form NH_3_ under the attack of H_ads_ produced from water splitting.^[^ ^30^ ^]^ Therefore, it is crucial to maintain a dynamic balance between high levels of H_ads_ production and consumption for efficient NH_3_ production. The regulation mechanism of H_ads_ in the NO_3_^−^RR process was studied using electron spin resonance (ESR) spectroscopy. In pure 0.1 m K_2_SO_4_, the DMPO‐H signal intensity was Cu‐CoP > CoP > Cu_3_P (Figure 4g), indicating that the H_ads_ were mainly generated at the Co site and promoting the water splitting process of CoP by doping with Cu elements.^[^ ^31^ ^]^ When NO_3_^−^ was added to the electrolyte, the DMPO‐H signal of CoP remained, while the DMPO‐H signal of Cu‐CoP was almost undetectable (Figure 4h), indicating the H_ads_ generated on the surface of Cu‐CoP was involved in the hydrogenation of nitrogen‐oxygen intermediates on the adjacent surfaces and was rapidly consumed. The projected density of states (PDOS) shows that the d‐band center of Cu‐CoP is closer to the Fermi level than CoP (Figure 4i), indicating that Cu‐doped CoP has a stronger interaction with H_ads_, which effectively inhibits H‐H dimerization and improves the Faraday efficiency of ammonia production from NO_3_^−^RR.^[^ ^32^ ^]^

**Figure 5:**
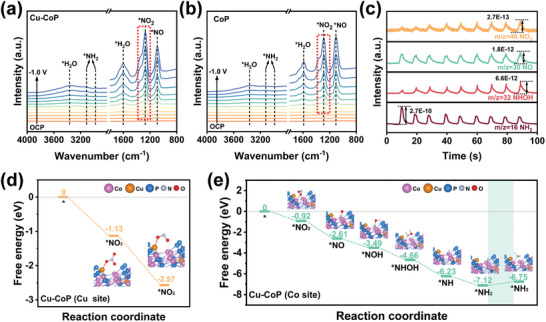
Electrochemical in situ FTIR spectra of a) Cu‐CoP and b) CoP during NO_3_^−^RR at different potentials; c) DEMS measurements of Cu‐CoP during NO_3_^−^RR; Free energy diagrams for the electrochemical reduction of nitrate to ammonia at the Cu d) and Co e) sites in Cu‐CoP.

In order to gain more insight into the catalytic process of NO_3_^−^ electroreduction to NH_3_ on Cu‐CoP, in situ electrochemical Fourier transform infrared spectroscopy (FTIR) was used to monitor the reaction process (**Figure** **5**a,b). For Cu‐CoP, characteristic peaks attributed to NO_2_^−^, NO, H_2_O, and NH_3_ were observed. The peak at ≈1090 cm^−1^ is attributed to the N─O bond, while the peak at 1272 cm^−1^ is attributed to the formation of ^*^NO_2_. The peaks at 3110 and 2977 cm^−1^ are attributable to ^*^NH_2_ intermediates, confirming the formation of NH_3_, while the peaks located at 3365 and 1601 cm^−1^ originate from the O─H stretching and bending modes of water, respectively, indicating the strong H_2_O splitting ability of Cu‐CoP.^[^ ^10^ ^]^ Meanwhile, the characteristic peaks of ^*^NO_2_ adsorption appeared at earlier potentials for Cu‐CoP compared to CoP, indicating that Cu‐CoP has a stronger NO_2_^−^ production capacity.^[^ ^26^ ^]^ Further exploration of the NO_3_^−^RR pathway by online differential electrochemical mass spectroscopy (DEMS) to probe reaction intermediates and products. As shown in Figure 5c and Figure S17 (Supporting Information), the detection of 46, 32, 31, 30, 17, 16, and 15 mass‐to‐charge ratio signals corresponds to NO_2_, NHOH, NOH, NO, NH_3_, NH_2_, NH. The reaction pathway for nitrate reduction on Cu‐CoP is tentatively deduced as ^*^NO_3_ → ^*^NO_2_ → ^*^NO → ^*^NOH → ^*^NHOH → ^*^NH → ^*^NH_2_ → ^*^NH_3_.

Differential charge density calculations were conducted to elucidate the electronic structure of the catalytically active sites, where yellow and cyan regions indicate charge accumulation and depletion, respectively. Figure S18 (Supporting Information) shows that the charge depletion of Cu atoms exhibits an electron‐deficient region, whereas the charge accumulation of adjacent Co and P atoms exhibits an electron‐rich region, indicating that the introduction of Cu element transfers the electrons from Cu to Co, induces charge redistribution, and the formation of positive and negative charge centers is conducive to the adsorption of NO_3_^−^ and H_ads_, respectively.^[^ ^33^ ^]^ The free energy profiles of the reaction intermediates for the initial ^*^NO_3_ to ^*^NO_2_ conversion at the Cu site and the subsequent ^*^NO_2_ to ^*^NH_3_ conversion at the Co site in Cu‐CoP are depicted in Figure 5d, e. These two processes with almost no thermodynamic energy barriers indicating that the Cu site has excellent NO_3_^−^ to NO_2_^−^ conversion ability and the Co site has excellent NO_2_^−^ reducing ability, the tandem catalytic interaction between the two sites enables the rapid NO_3_^−^RR occurrence. Furthermore, the RDS of this tandem catalytic pathway is the ^*^NH_2_ hydrogenation process (ΔG = 0.37 eV), and the H_ads_‐rich environment on the catalyst surface is favorable to facilitate this step.^[^ ^34^ ^]^ The tandem reaction mechanism of Cu‐CoP is strongly demonstrated based on experiments, in situ characterization, and theoretical calculations. The NO_3_^−^ is preferentially adsorbed on the Cu sites and reduced to NO_2_^−^, which is subsequently desorbed into the electrolyte and adsorbed to the Co sites for the subsequent hydrogenation process.

## 3 Conclusion

In summary, we proposed a Cu‐Co dual sites tandem synergistic effect by constructing Cu doped CoP to facilitate the adsorption and conversion of NO_3_^−^ on Cu and to accelerate the water splitting and optimize H_ads_ adsorption on CoP leading to the significantly high NO_3_^−^RR performance. The designed Cu‐doped CoP catalysts exhibit excellent electrocatalytic activity in the reduction of neutral low concentrations (10 mm) of NO_3_^−^ to NH_3_, achieving a significant NH_3_ yield of 7.68 mg h^−1^ cm^−2^ with an 84.8% FE at −1.0 V, which is the highest for NH_3_ production from NO_3_^−^RR at neutral low concentration. The doping of Cu into CoP effectively couples the unique catalytic activities of Cu and Co for the reduction of NO_3_^−^ to NO_2_^−^ and NO_2_^−^ to NH_3_, respectively, and completes the NO_3_^−^RR production of NH_3_ through a tandem catalytic process. In situ tests and theoretical calculations indicated that the introduction of Cu element could significantly enhance the adsorption of NO_3_^−^ and the generation rate of the reaction intermediate NO_2_^−^, providing enough NO_2_^−^ supply for the subsequent reduction of NO_2_^−^ to NH_3_ at the Co site, thus promoting the reduction of NO_3_^−^ to NH_3_. Furthermore, the modulation of the electronic structure of Co by Cu promoted water splitting and optimized adsorption of H_ads_ to prevent H_2_ release. This work not only provides a promising catalyst for the efficient production of NH_3_ from NO_3_^−^RR at neutral low concentrations, but also provides insights into improving the catalytic performance by regulating the dynamic equilibrium of H_ads_ in tandem catalytic systems.

## Conflict of Interest

The authors declare no conflict of interest.

## Author Contributions

W.Y. and Z.C. contributed equally to this work. W.Y. and X.C. conceived and designed the experiment. X.C., L.W., and J.S. provided funding support and revisions to the manuscript. W.Y. and Z.C. did the experiments and wrote the manuscript. Y.X., P.W., and R.S. helped with experiments and characterization. All authors discussed the findings and commented on the manuscripts.

## Supporting information



Supporting Information

## Data Availability

The data that support the findings of this study are available from the corresponding author upon reasonable request.
