# Supplementary material for: Cu‐Co Dual Sites Tandem Synergistic Effect Boosting Neutral Low Concentration Nitrate Electroreduction to Ammonia
Source: Adv Sci (Weinh). 2025 Feb 17;12(14):2416386. doi: 10.1002/advs.202416386 (PMC11984843; doi:10.1002/advs.202416386)
Supplement: Supplementary file 1 — Supporting Information [file ADVS-12-2416386-s001.docx]

**Cu-Co dual sites tandem synergistic effect boosting neutral low-concentration nitrate electroreduction to ammonia**

Wenhao Yang^1,2+^, Ziwei Chang^3+^, Xu Yu^1,2^, Ping Wu^1^, Ruxiang Shen^1^, Lianzhou Wang^4^, Xiangzhi Cui^1,2,5*^, Jianlin Shi^1,2^

^1^ State Key Lab of High Performance Ceramics and Superfine Microstructure, Shanghai Institute of Ceramics, Chinese Academy of Sciences, Shanghai 200050, PR China

^2^ Center of Materials Science and Optoelectronics Engineering, University of Chinese Academy of Sciences, Beijing 100049, PR China

^3^ School of Physical Science and Technology, Shanghai Tech University, Shanghai 201210, PR China

^4^ Nanomaterials Centre, School of Chemical Engineering and Australian Institute for Bioengineering and Nanotechnology, the University of Queensland, St Lucia, QLD 4072, Australia

^5^ School of Chemistry and Materials Science, Hangzhou Institute for Advanced Study, University of Chinese Academy of Sciences, Hangzhou 310024, PR China

*Email: cuixz@mail.sic.ac.cn (X. Cui).

1. **Experimental Section**

**1.1 Materials**

Cobaltous nitrate hexahydrate (Co(NO_3_)_2_·6H_2_O, ≥98.5%), Copper nitrate trihydrate (Cu(NO_3_)_2_·3H_2_O, ≥99%), Potassium nitrate (KNO_3_, ≥99.0%), Phosphoric acid (H_3_PO_4_, 85%) Sulfuric acid (H_2_SO_4_) and Hydrochloric acid (HCl) were purchased from Chinese medicine reagent. 2-Methylimidazole (C_4_H_6_N_2_, 98%), Deuterium oxide (D_2_O, 99.9%), Maleic acid (C_4_H_4_O_4_, 99%), Potassium bicarbonate (KHCO_3_, ≥99.5%), Potassium sulphate (K_2_SO_4_, ≥99%), Potassium nitrite (KNO_2_, 97%), Sulfamic acid (NH_3_SO_3_, 99%), p-aminobenzenesulfonamide (C_6_H_8_N_2_O_2_S, 99%), N-(1-naphthyl) ethylenediamine dihydrochloride (C_12_H_16_Cl_2_N_2_, 99%) and Sodium nitroprusside dihydrate (Na_2_[Fe(CN)_5_NO]·2H_2_O, 99%) were purchased from Adamas Reagent Co. Ltd (Tansoole). Sodium salicylate (C_7_H_5_NaO_3_, ≥99.5%), Potassium sodium tartrate tetrahydrate (KNaC_4_H_4_O_6_·4H_2_O, ≥99%), Sodium hypochlorite (NaClO, available chlorine≥7.5%), and Ammonium chloride (NH_4_Cl, ≥99.9%) were purchased form Greagent (Tansoole). Sodium hypophosphite (NaH_2_PO_2_), Ammonium-^14^N sulfate ((^14^NH_4_)_2_SO_4_, ≥99.0%), Ammonium -^15^N sulfate ((^15^NH_4_)_2_SO_4_, 99 atom %, ≥98.5%), Sodium nitrate-^15^N (Na^15^NO_3_, 99atom%; ≥98.5%) and 5,5-Dimethyl-1-pyrroline N-oxide (C_6_H_11_NO, 97%) were all purchased from Shanghai Aladdin Biochemical Technology Co. Ltd. All chemicals were used as received without any further purification.

- 1. **Materials preparation**

**Synthesis of ZIF-L (Co) precursor on Nickel foam (NF)**

First, the NF (2×3 cm^2^) was sonicated in 3 M HCl for 15 min to remove its surface oxide layer. Next, the NF was sonicated with water, ethanol and water in turn for 5 min each to remove the surface contaminants and then dried in the vacuum oven at 60 ℃. 40 ml 0.4 M 2-Methylimidazole (C_4_H_6_N_2_) aqueous solution was quickly poured into 40 ml 0.05 M cobalt nitrate hexahydrate (Co(NO_3_)_2_·6H_2_O) aqueous solution, then the dried NF was added. After stirring for 4h, NF was clipped out and rinsed with deionized water to remove excess ZIF-L(Co) from the surface, and then dried in the vacuum oven at 60 °C to obtain the ZIF-L(Co)@NF.

**Synthesis of Cu-CoP on NF**

ZIF-L(Co)@NF was used as the working electrode, the graphite rod as the counter electrode, Ag/AgCl as the reference electrode, and an aqueous solution containing 0.1 M KHCO_3_ and 3 mM of Cu(NO_3_)_2_·3H_2_O was used as the electrolyte, and argon was passed through it for 5 min before applying the voltage in order to remove the oxygen from the solution. A constant voltage pulse strategy was used for cation exchange to reach a target value of 1 C cm^-2^ charge at potentials of -0.7 V vs. Ag/AgCl and -0.9 V vs. Ag/AgCl, respectively, and the process was repeated until a total charge of 4 C cm^-2^ was reached. During the synthesis process, some of Co ions were dissolved from the structure of ZIF-L(Co) by the combined effect of an applied potential of -0.7 V vs. Ag/AgCl and a weakly alkaline KHCO_3_ solution, which produced cationic vacancies on the ZIF-L(Co) surface. With the subsequent applied potential of -0.9 V vs. Ag/AgCl, Cu ions and Co ions will fill the cationic vacancies for redeposition to realize Cu doping.

The prepared material was rinsed with deionized water and dried in a vacuum oven at 60 °C for 12 h. The dried material and 0.5 g of sodium hypophosphite were placed in two ceramic boats, where the sodium hypophosphite was placed upstream of the tube furnace. The temperature in the center of the tube furnace was increased to 350 °C at a heating rate of 2 °C min^-1^ under Ar gas atmosphere and maintained for 2 h, and then naturally cooled to room temperature to obtain Cu-doped cobalt phosphide catalyst (Cu-CoP).

- 1. **Materials characterization**

Scanning electron microscope (SEM) images were taken with Thermo Quattro S field emission environmental scanning electron microscope. Transmission electron microscopy (TEM) images and corresponding energy-dispersive X-ray spectrometer mapping (EDS-mapping) were acquired on a FEI Talos F200S field emission transmission electron microscope (200 kV). The powder X-ray diffraction (XRD) was recorded at 2^o^ min^-1^ on a Rigaku D/Max-2550 V X-ray diffractometer with a Cu Kα radiation target (40 KV, 30 mA). X-ray photoelectron spectroscopy (XPS) was collected using a Thermo Scientific K-Alpha XPS spectrometer. Ultraviolet-visible (UV-Vis) absorbance spectra were measured on a UV-1900i Spectrophotometer. Nuclear magnetic resonance (NMR) spectrometer was detected on an Avance Ⅱ 500 instruments (Bruker, 600 MHz). The differential electrochemical mass spectrometry (DEMS, QAS 100) was provided by Linglu instruments (Shanghai) Co. Ltd. In situ Fourier transform infrared spectroscopy (FTIR) was conducted on Nicolet Nexus 670 Spectroscopy system. Electron spin resonance (ESR) was carried out by a Bruker EMXnano instrument. The X-ray absorption spectroscopy (XAS) spectra were recorded in the transmission mode at the BL14W1 beamline of Shanghai Synchrotron Radiation Facility (SSRF).

- 1. **Electrochemical measurements**

All NO_3_^-^RR electrochemical performances were measured on a CHI 760E electrochemical workstation (CH instruments, Inc., Shanghai). Ag/AgCl electrode and a graphite rod were used as reference and counter electrodes, respectively, at room temperature. Ag/AgCl electrode was stocked in a saturated KCl solution and washed with deionized water before use. All potentials were calibrated to the reversible hydrogen electrode (RHE) scale by the Nernst equation (E_RHE_ = E_Ag/AgCl_ + 0.0591pH + 0.1989 V). The electrochemical NO_3_^-^RR measurements were acquired in 0.1 M K_2_SO_4_ with and without 10 mM KNO_3_ solution by using an H-type electrolytic cell with the cathode and anode chambers separated by a FAB-PK-130 membrane. The linear sweep voltammetry (LSV) test were carried out in a three-electrode system at scanning rates of 50 mV s^-1^. EIS measurements were conducted in a frequency range from 10^5^ to 0.01 Hz with 5 mV amplitude. During stability test, the electrolyte was changed approximately every 12 hours without changing the working electrode and FAB-PK-130 membrane.

- 1. **Determination and quantitation of ammonia using UV–vis**

We used the sodium salicylate method to detect the ammonia concentration. In detail, a quantity of electrolyte was taken from the electrolytic cell after the NO_3_^-^RR and diluted to 2 mL, to which were added 2 mL of sodium salicylate solution (80 g l^−1^ NaOH, 50 g l^-1^ sodium salicylate and 50 g l^-1^ potassium sodium tartrate), 0.2 mL of sodium nitroprusside solution (10 g l^-1^ sodium nitroprusside), and 1 ml of sodium hypochlorite solution (35 ml l^-1^ sodium hypochlorite, available chlorine ≥7.5 %), respectively. After 60 min, the absorption spectra of aforementioned solutions were tested using a UV-Vis spectrophotometer and the absorption intensity at 655 nm was recorded. In the same manner as described above, a concentration-absorbance curve was constructed by adding a series of standard ammonium chlorides to a 0.1 M K_2_SO_4_ solution to accurately quantify ammonia. To make a background correction, the absorbance of all samples tested was subtracted from the absorbance of the sample without ammonia.

- 1. **Determination and quantitation of nitrite using UV–vis**

We used the Griess test to detect the nitrite concentration. The Griess reagent consisted of 40 g l^-1^ *p*-aminobenzenesulfonamide, 2 g l^-1^ N-(1-naphthyl)-ethylene-diamine dihydrochloride and 100 ml l^-1^ phosphoric acid. Take a quantity of electrolyte from the electrolytic cell after NO_3_^-^RR and dilute it to 5 ml, to which were added 0.1 mL of Griess reagent. After 20 min, the absorption spectra of aforementioned solutions were tested using a UV-Vis spectrophotometer and the absorption intensity at 540 nm was recorded. In the same manner as described above, a concentration-absorbance curve was constructed by adding a series of standard potassium nitrite to a 0.1 M K_2_SO_4_ solution to accurately quantify nitrite.

**1.7 Determination and quantitation of nitrate using UV–vis**

Initially, a predefined volume of the sample was diluted to 5 mL within the detection limit. To this solution, 0.1 mL of 1 M hydrochloric acid and 0.01 mL of a 0.8 wt% solution of sulfamic acid were added. After holding for 15 minutes, measure the absorption spectrum using a UV-Vis spectrophotometer and record the net absorbance value (A = A_220 nm_ - 2A_275 nm_). In the same manner as described above, a concentration-absorbance curve was constructed by adding a series of standard potassium nitrate to a 0.1 M K_2_SO_4_ solution to accurately quantify nitrite.

**1.8 Isotope labeling experiment and ^1^H NMR quantitative experiment**

Take 5 mL of electrolyte from the electrolytic cell after NO_3_^-^RR and adjust the pH to 2 by adding 0.1 ml of 0.3 M sulfuric acid. Subsequently, 0.45 ml of mixed solution, 50  μl of Maleic acid solution (0.1 g maleic acid dissolved in 25 mL D_2_O) were transferred to the NMR tube. In the same manner as described above, a concentration-peak area curve was constructed by adding a series of standard ammonium chloride to a 0.1 M K_2_SO_4_ solution to accurately quantify nitrite.

**1.9 Calculation of NH_3_ yield and Faradaic efficiency**

The ammonia yield was calculated using the following equation:

$$Y_{{NH}_{3}} \left( mg h^{-1}{cm}^{-2} \right)=\frac{c \left( ug {mL}^{-1} \right)\times V \left( mL \right)\times17 (g {mol}^{-1})}{14 (g {mol}^{-1})\times t \left( h \right)\times A \left( {cm}^{2} \right) \times1000}$$

Where $c$is the detected NH_4_^+^-N concentration, $V$is the volume of the electrolyte, $t$is the reduction reaction time, $A$is the geometric area of the working electrode.

The Faradaic efficiency was determined with the following equation:

$${FE}_{{NH}_{3}}\left( \% \right)=\frac{8\times F \left( C {mol}^{-1} \right)\times C \left( \mu g {mL}^{-1} \right)\times V(mL)}{14 (g {mol}^{-1})\times Q (C)\times{10}^{6}}$$

Where: $F$is Faraday constant (96485 C mol^-1^), $M$is the relative molecular mass of N, $Q$ is the total charge during the reaction.

**1.10 Computational details**

All spin-polarized DFT calculations in this chapter were performed using the VASP software, and the PAW method was used to describe the interactions between valence electrons and ions^[1]^. The generalized gradient approximation function proposed by Perdew, Burke and Ernzerhof^[2]^ was chosen for the exchange correlation generalization of the system^[3]^. The plane-wave truncation energy is set to 500 eV. the energy convergence criterion is set to 10^-5^ eV in the iterative solution of the Kohn-Sham equation. a vacuum layer of 15 Å is added in the direction perpendicular to the model to avoid artificial interactions between the periodic structures. All calculations were performed using a 2 × 2 × 1 K-point grid for Brillouin zone integration, and all structures were fully relaxed until the residual forces on the atoms decreased to less than 0.02 eV/Å^[4]^. The DFT-D3 semiempirical corrections were carried out by the Grimme method^[5]^, and the change in the Gibbs free energy (∆G) for each elemental step was obtained from Eq. 4.4:

$G=\Delta E+\Delta ZPE-T\Delta S+\Delta G_{U}+\Delta G_{pH}$ (1)

where ∆E and ∆ZPE are the adsorption energy calculated from the density functional theory and the adsorption energy obtained from the zero-point energy correction, respectively. T, ∆S, U (U = 0), and ∆G_pH_ represent the temperature, entropy difference, the applied electrode potential, and pH free energy corrections, respectively.

1. **Supporting** **figures**


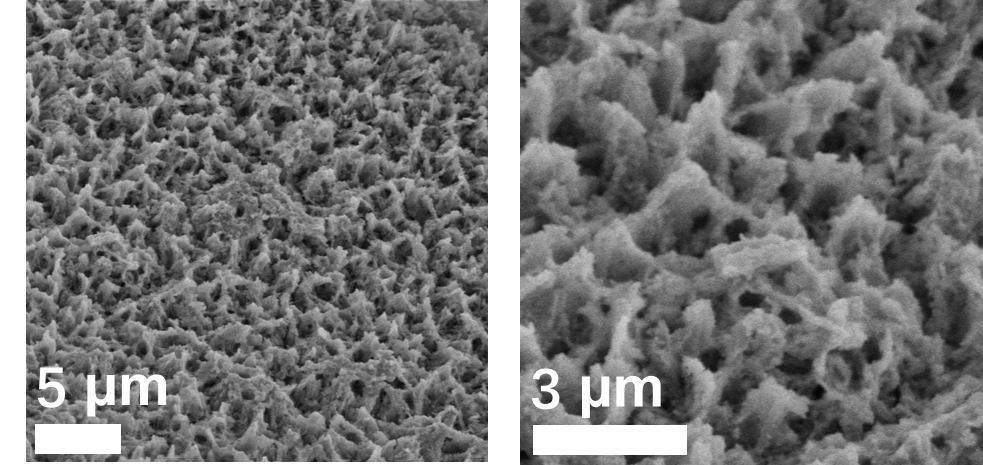


**Figure S1.** SEM images of ZIF-L(Co) after ED-CE processing at different magnifications.


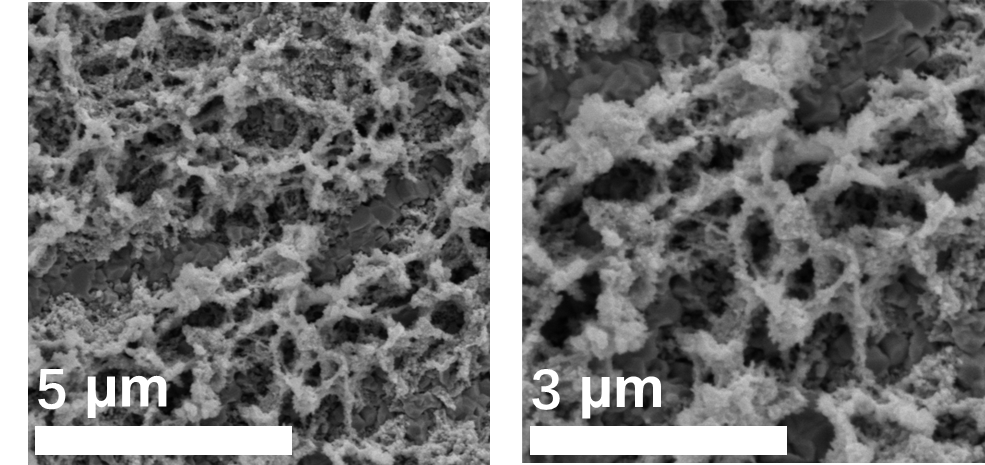


**Figure S2.** SEM images of Cu-CoP at different magnifications.


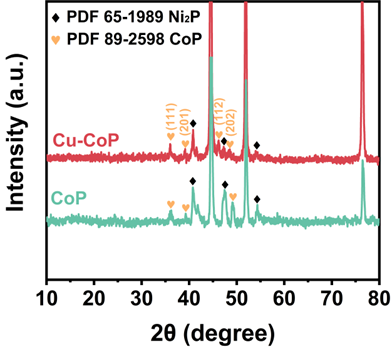


**Figure S3.** XRD patterns of CoP and Cu-CoP.


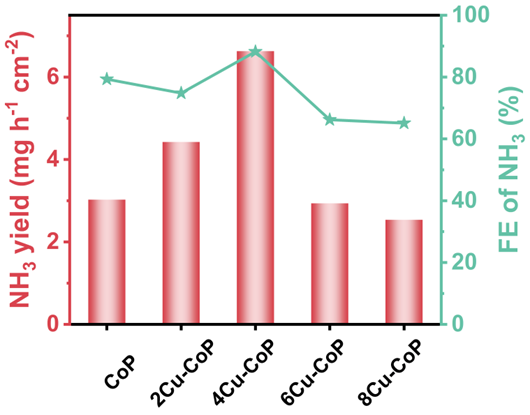


**Figure S4.** NH_3_ yield and FE of Cu-CoP synthesized at different charges.


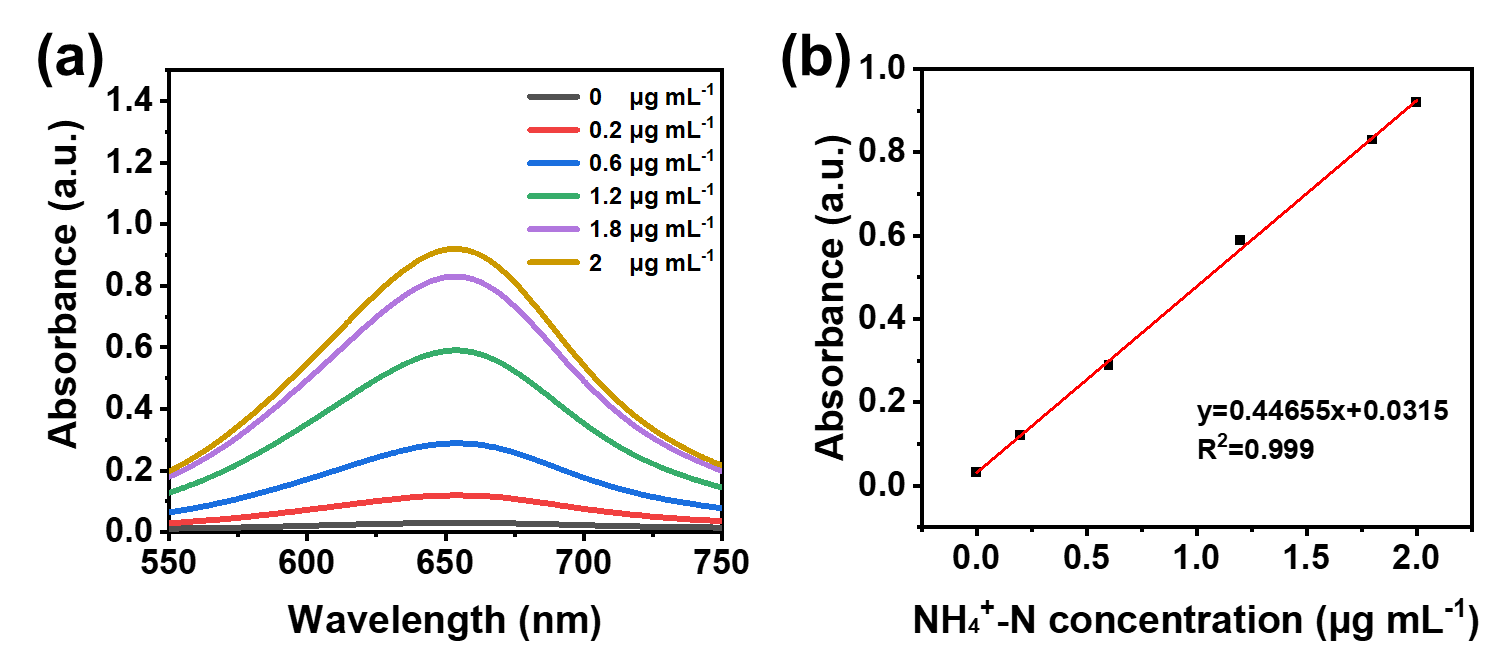


**Figure S5.** Calibration curve in 0.1 M K_2_SO_4_ using ammonium chloride solutions of known concentration as standards. (a) Spectrophotometric UV-vis curves of salicylic acid after incubated for 1 hours and (b) calibration curve used for the estimation of N-NH_4_^+^ concentration. The absorbance at 655 nm was measured by a UV-Vis spectrophotometer, and the fitting curve shows good linear relation of absorbance with N-NH_4_^+^ concentration (y = 0.446x + 0.0315, R^2^=0.999).


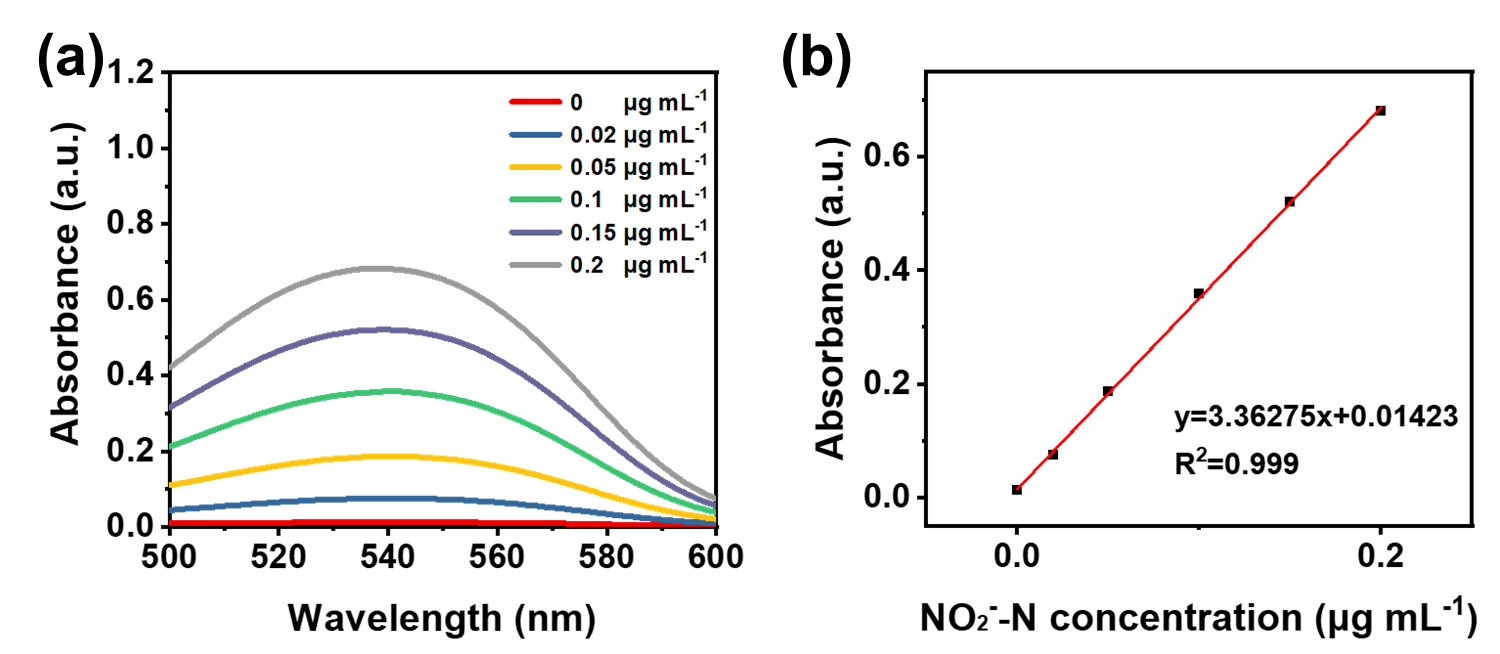


**Figure S6.** Calibration curve in 0.1 M K_2_SO_4_ using potassium nitrite solutions of known concentration as standards. (a) Spectrophotometric UV-vis curves of Griess test after incubated for 20 minutes and (b) calibration curve used for the estimation of N-NO_2_^-^ concentration. The absorbance at 540 nm was measured by a UV-Vis spectrophotometer, and the fitting curve shows good linear relation of absorbance with N-NO_2_^-^ concentration (y = 3.36x + 0.0142, R^2^=0.999).


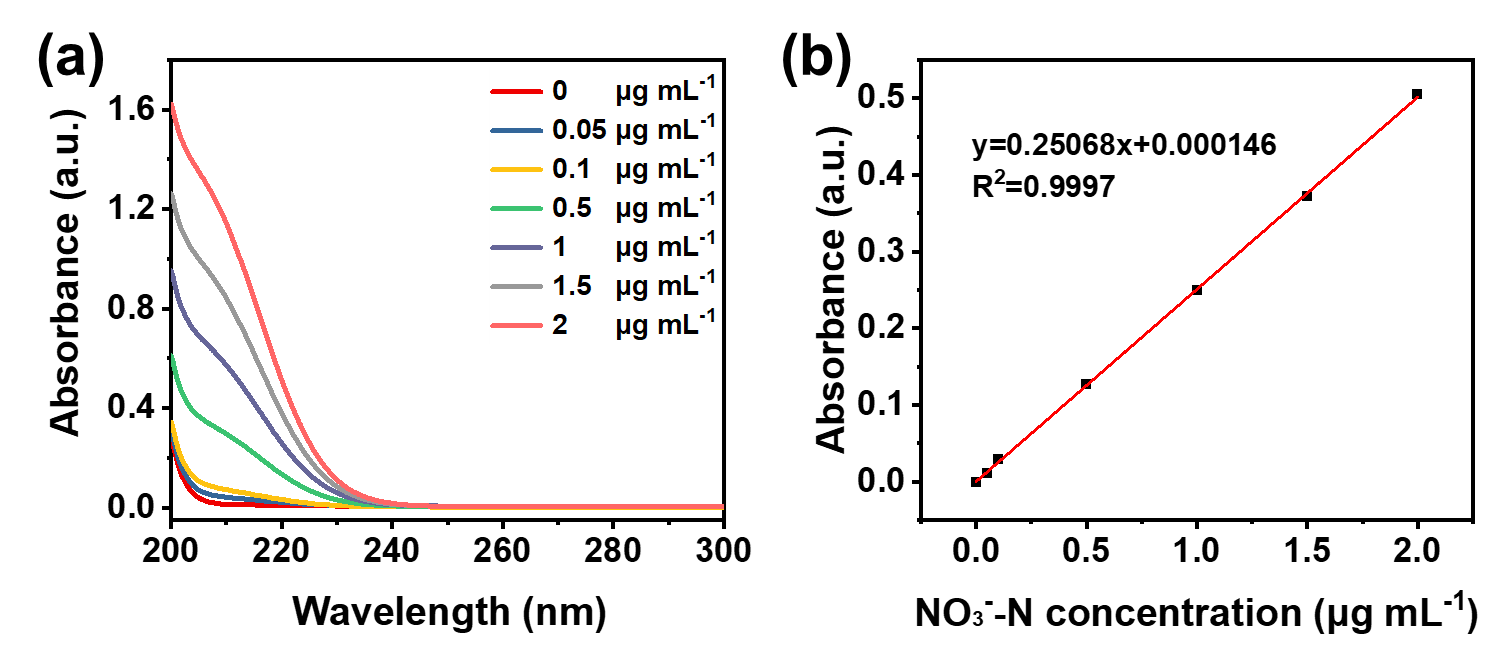


**Figure S7.** Calibration curve in 0.1 M K_2_SO_4_ using potassium nitrite solutions of known concentration as standards. (a) Spectrophotometric UV-Vis curve at 20 min after addition of detection reagent and (b) Calibration curve for estimation of N-NO_3_^-^ concentration. The absorbance at 275 and 220 nm were measured by UV-visible spectrophotometer, and the fitting curve showed a good linear relationship between absorbance (A=A_220nm_-2A_275nm_) and N-NO_3_^-^ concentration.


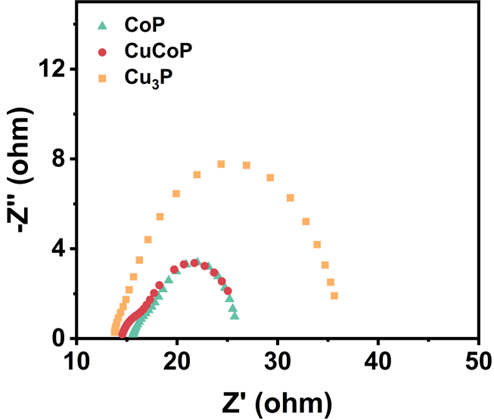


**Figure S8.** EIS for Cu-CoP, CoP and Cu_3_P in a frequency range from 10^5^ to 0.01 Hz with 5 mV amplitude.


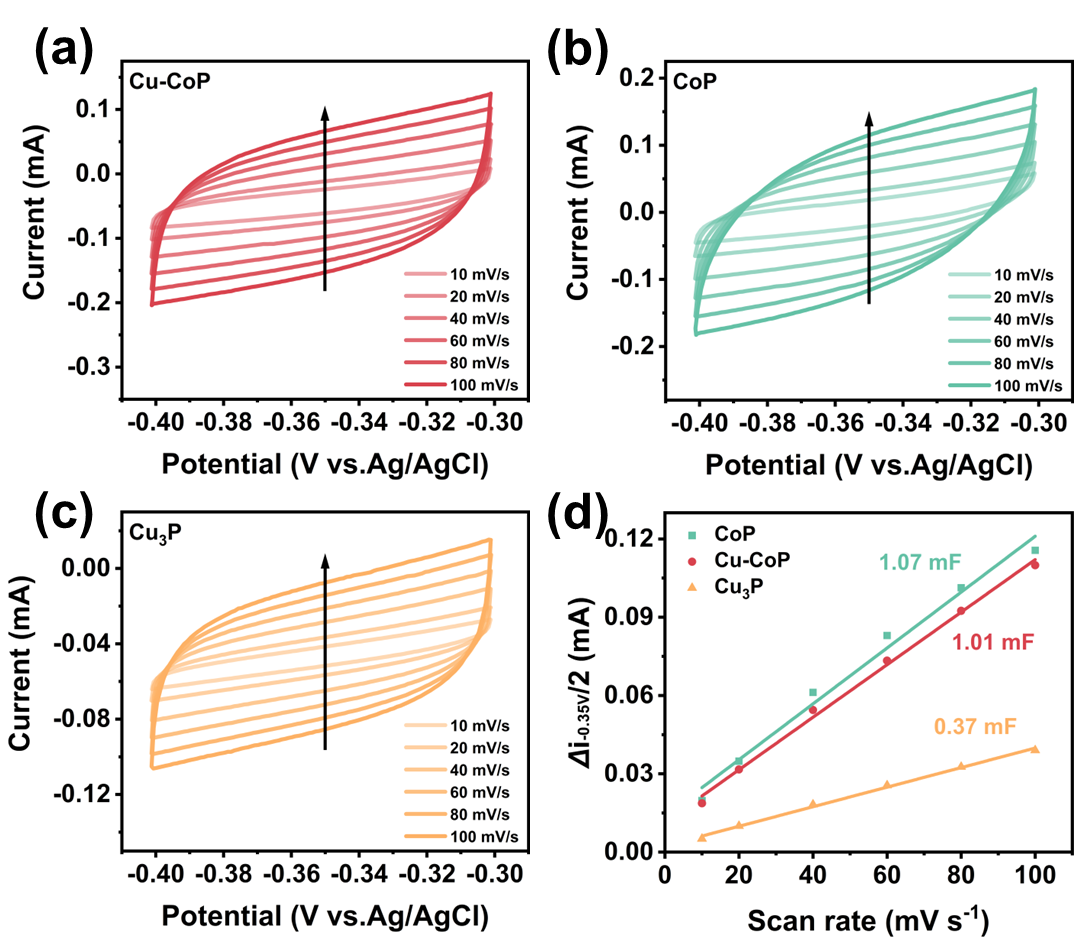


**Figure S9.** CV curves of Cu-CoP (a), CoP (b) and Cu_3_P (c)at varied scan rates (10 to 100 mV s-1) in the region of -0.4 to -0.3 V (vs. Ag/AgCl). Electrochemical double layer capacitances (Cdl) for Cu-CoP, CoP and Cu_3_P.


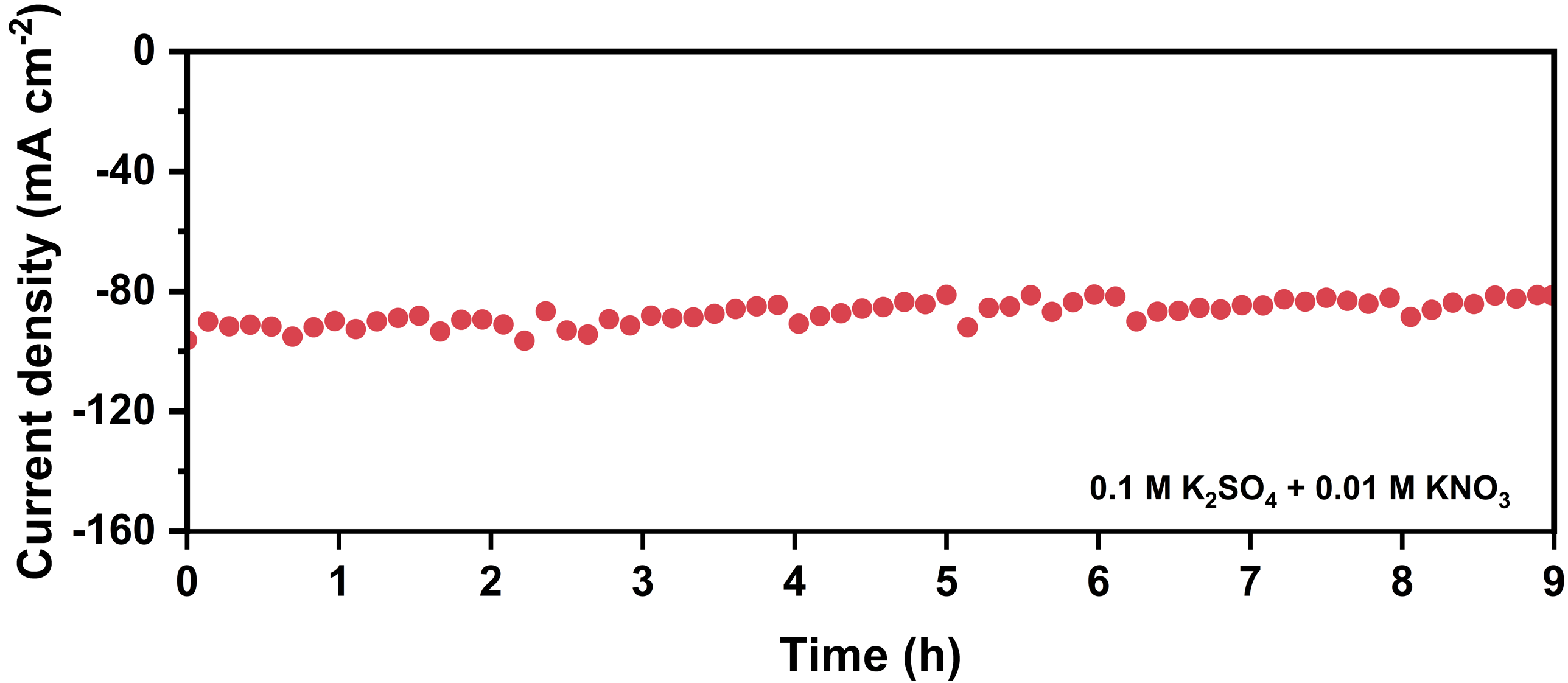


**Figure S10.** Long-time stability test of Cu-CoP catalyst at -0.8 V (vs. RHE) after the electrolytic cell connected to the circulating electrolyte.


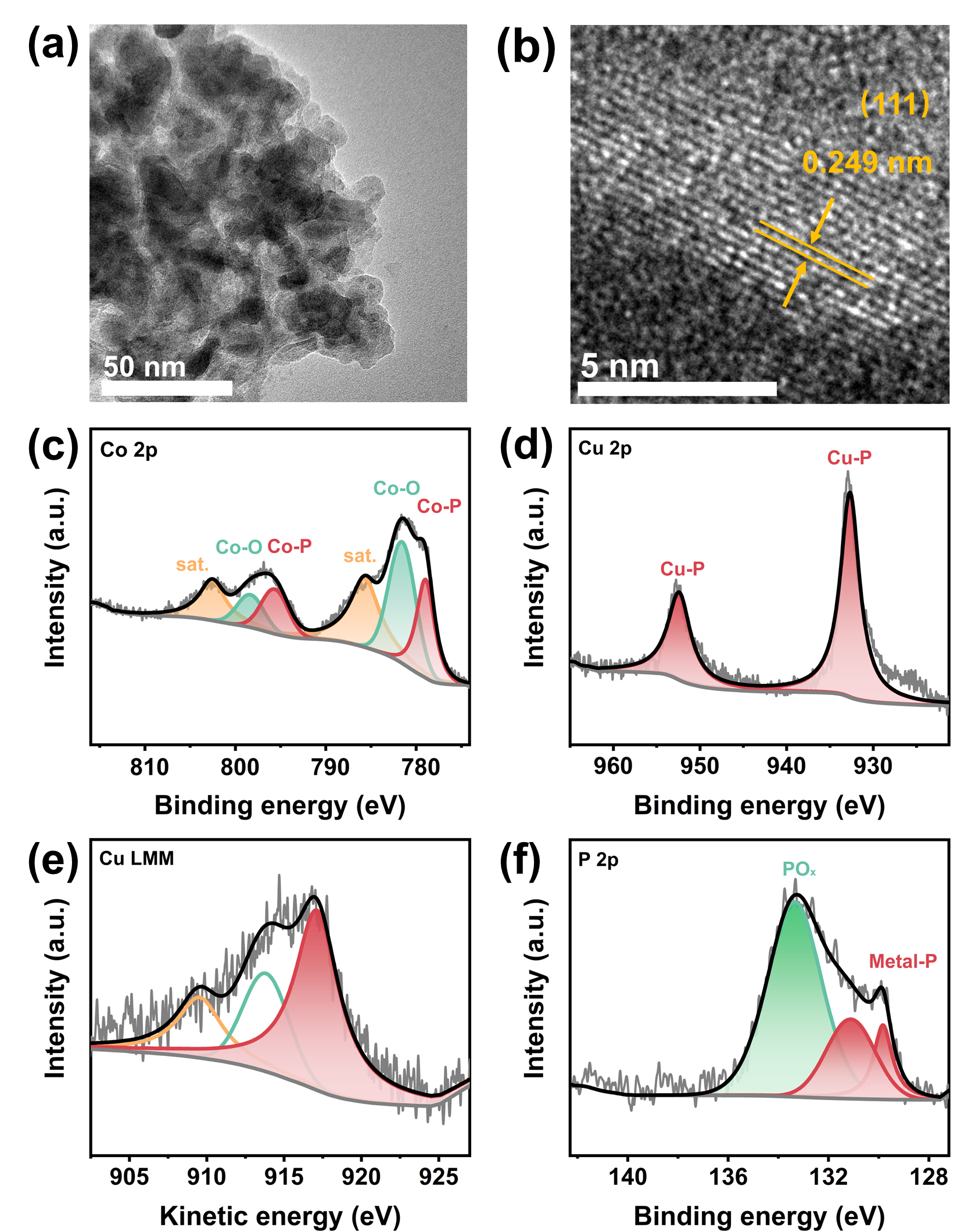


**Figure S11.** Characterizations of Cu-CoP after the stability testing. (a) TEM, (b) HRTEM; XPS spectra of Co 2p (c), Cu 2p (d) and P 2p (f), Cu LMM AES spectra (e).


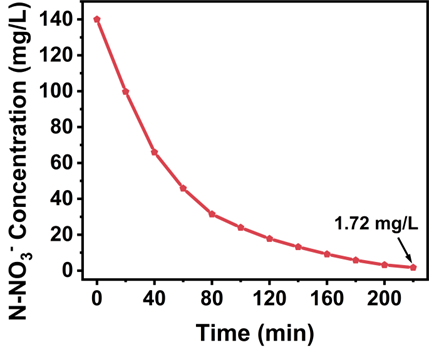


**Figure S12.** Curves of NO_3_^-^ consumption by Cu-CoP catalyst at an applied potential of -0.8 V (vs. RHE).


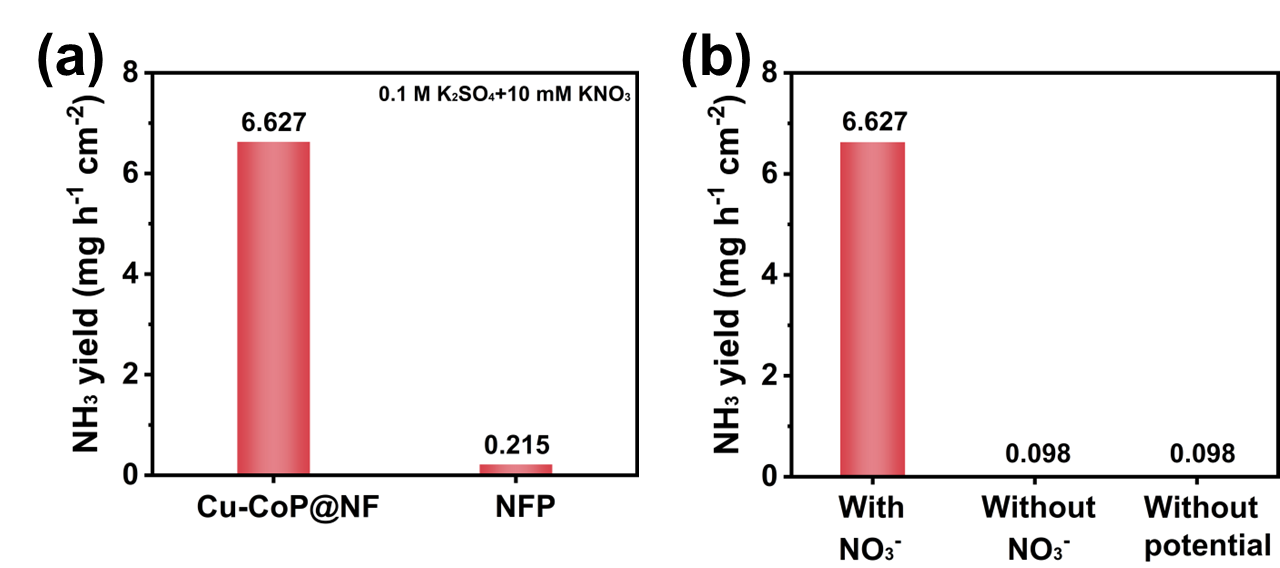


**Figure S13.** (a) Ammonia yield of Cu-CoP and phosphated nickel foam substrates at a potential of -0.8 V, (b)NH_3_ yields of Cu-CoP at different conditions.


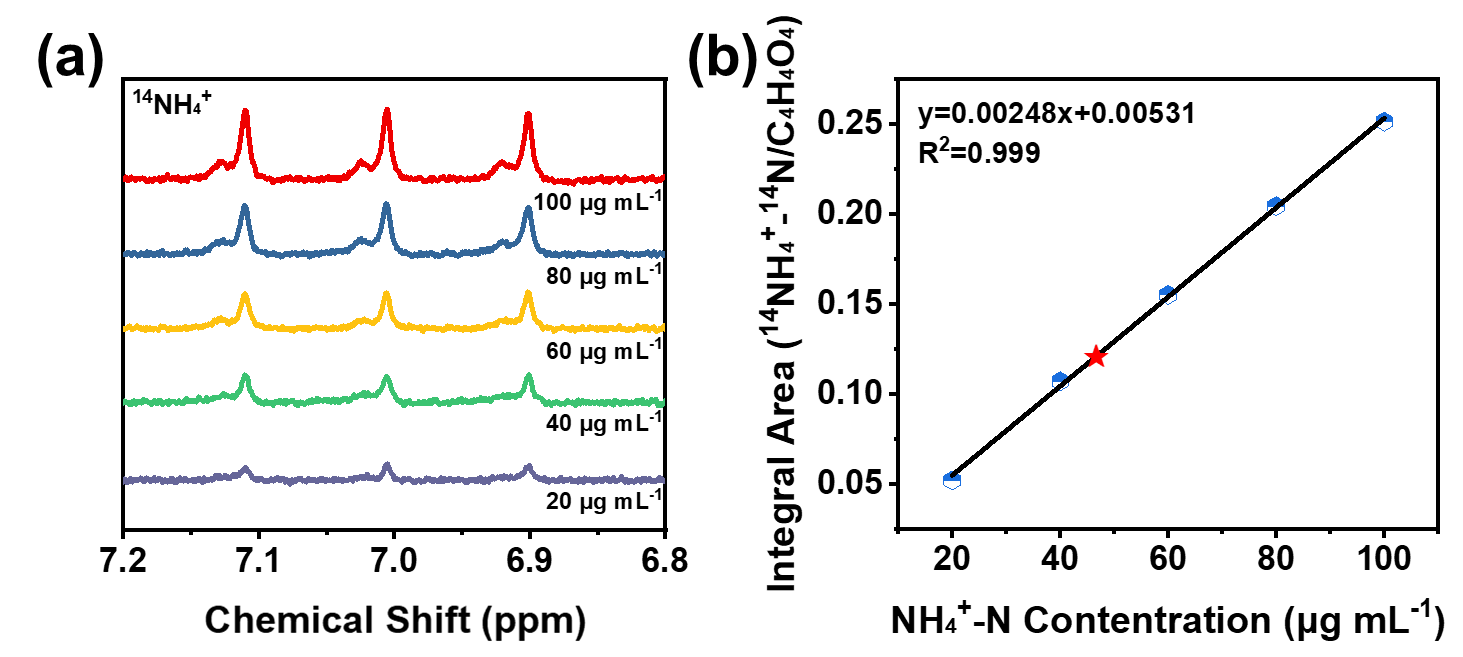


**Figure S14.** (a) ^1^H NMR spectra (600 MHz) and (b) calibration curve used for the estimation of ^14^N-NH_4_^+^ concentration.


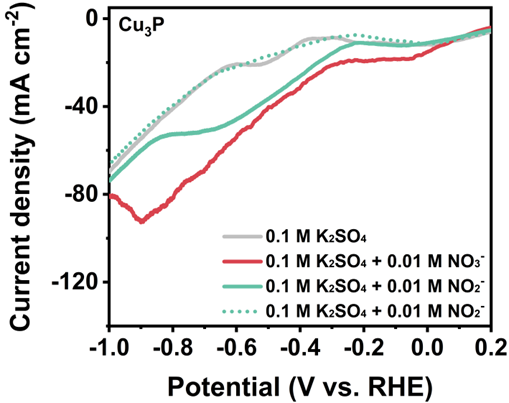


**Figure S15.** The LSV of Cu_3_P catalysts in different electrolytes, the green dashed line indicates the second LSV carried out in an electrolyte containing 0.01 M NO_2_^-^ electrolyte.


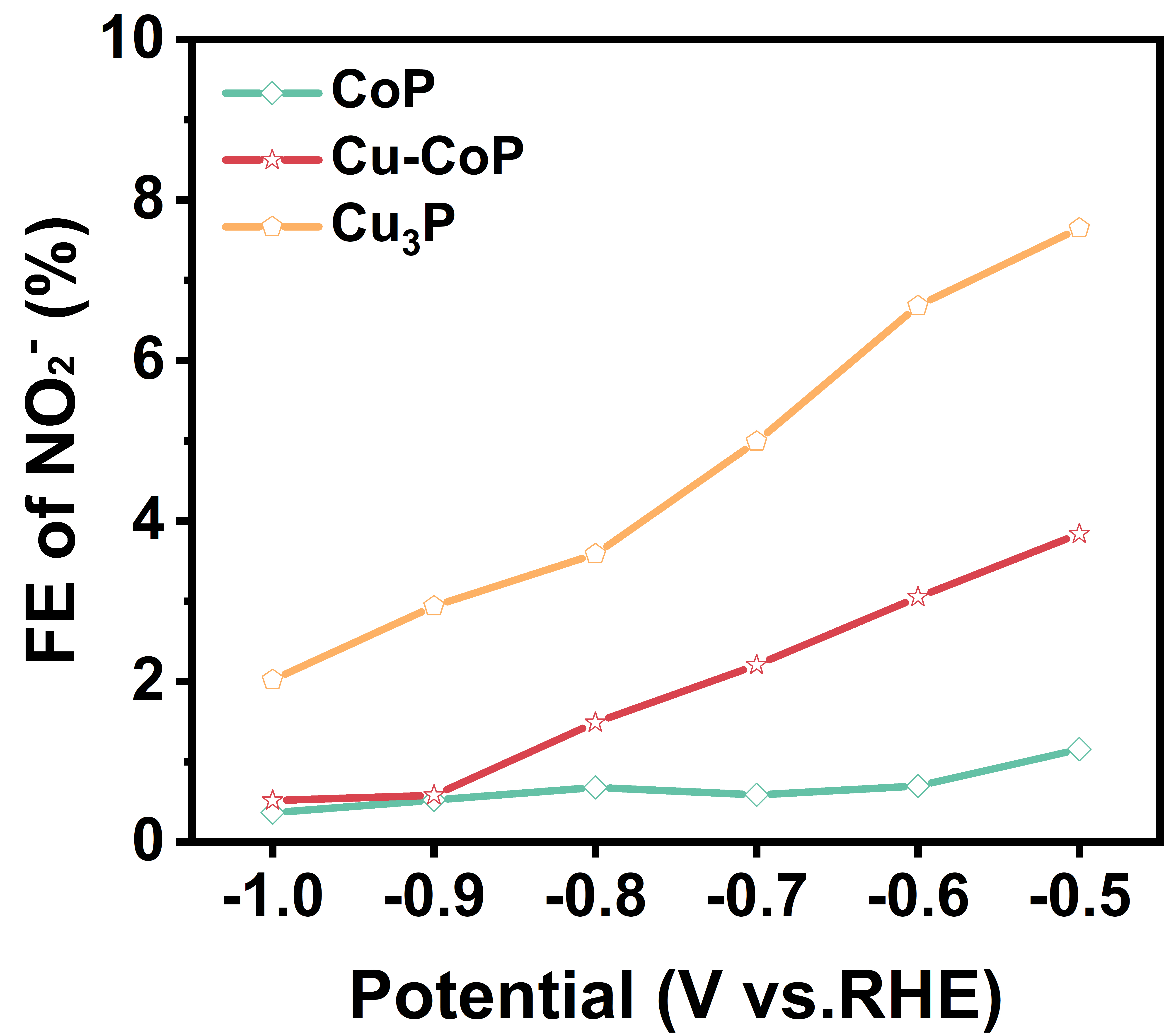


**Figure S16.** CoP, Cu-CoP and Cu_3_P at different applied potentials for NO_3_^-^ to NO_2_^-^ for FE.


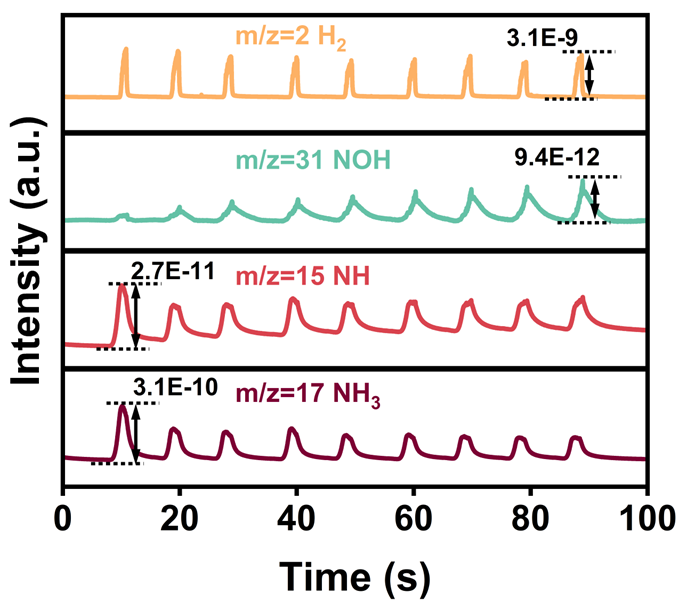


**Figure S17.** DEMS measurements of Cu-CoP during NO_3_^-^RR.


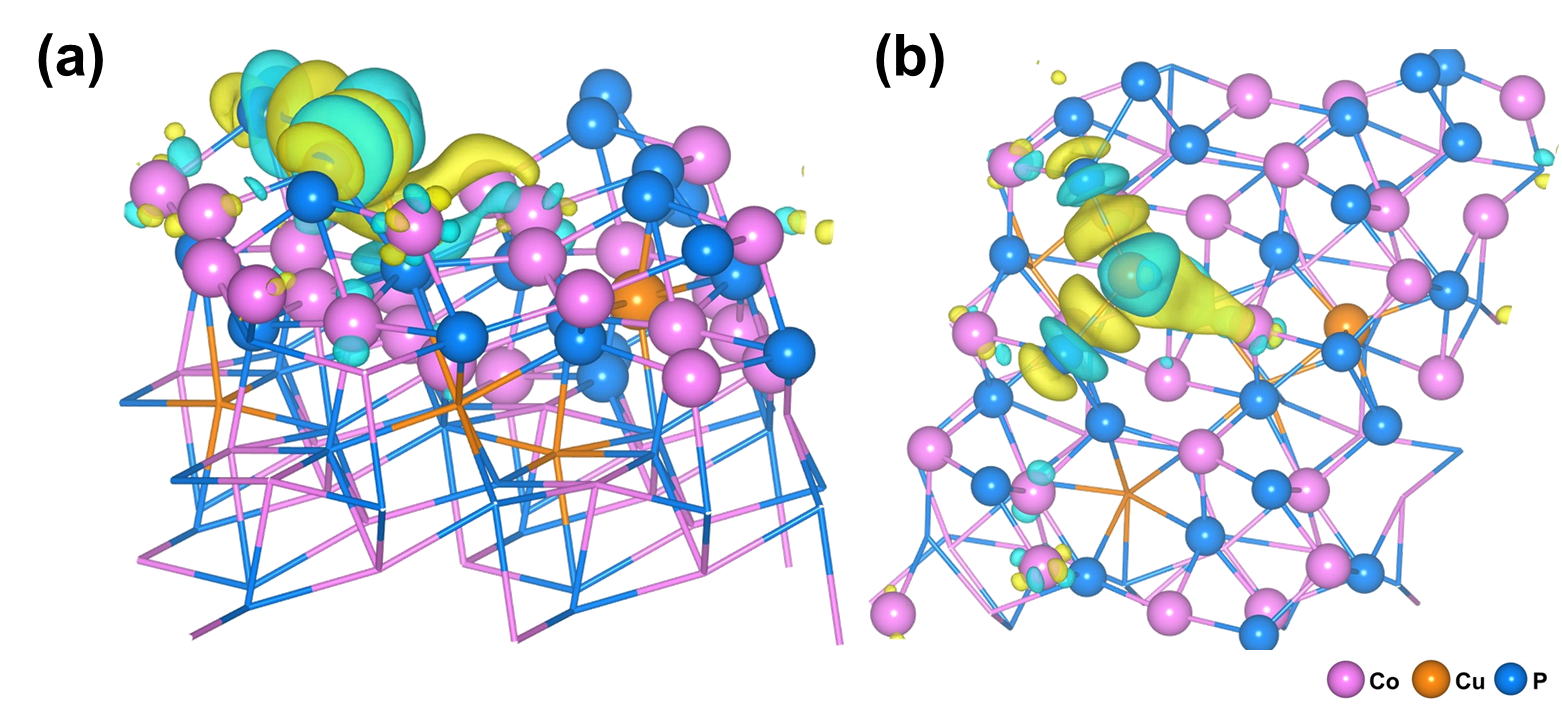


**Figure S18.** The main (a) and top (b) views of the calculated Cu-CoP charge density difference.

**Supplementary Table S1** Performance comparisons of Cu-CoP catalyst with previously reported electrocatalysts for NO_3_^-^RR

| Catalysts | Electrolyte | Potential  (V vs. RHE) | NH_3_ yield | FE  （%） | Reference |
| --- | --- | --- | --- | --- | --- |
| CoCuP | 0.1 M K_2_SO_4_ +  10 mM KNO_3_ | -0.5  -1.0 | 2.91 mg h^-1^ cm^-2^  7.65 mg h^-1^ cm^-2^ | 89.2  85.1 | This work |
| 1T-MoS_2_ | 0.5 M K_2_SO_4_ +  10 mM KNO_3_ | -0.5 | 0.653 mg h^-1^ cm^-2^ | 88.12 | ^[6]^ |
| CuCoAl LDH | 0.5 M PBS +  0.05 M KNO_3_ | -0.8 | 3.74 mg h^-1^ mg_cat_^-1^ | 99.5 | ^[7]^ |
| Cu-PTCDA | 0.1 M PBS +  8.06 mM KNO_3_ | -0.4 | 0.436 mg h^-1^ cm^-2^ | 85.9 | ^[8]^ |
| Cu/Cu_2_O | 0.5 M Na_2_SO_4_  14.3 mM NaNO_3_ | -0.85 | 4.158 mg h^-1^ cm^-2^ | 95.8 | ^[9]^ |
| Disordered RuO_2_ | 0.5 M Na_2_SO_4_ +  14.3 mM NaNO_3_ | -0.35 | 1.97 mg h^-1^ cm^-2^ | 97.5 | ^[10]^ |
| Fe@Cu_1_FeO_x_ | 0.1 M K_2_SO_4_ +  71.4 mM KNO_3_ | -1.3V vs.  SCE | 1.98 mg h^-1^ cm^-2^ | 95.4 | ^[11]^ |
| CuCoSP | 0.1 M KOH +  10 mM KNO_3_ | -0.175 | 2.64 mg h^-1^ cm^-2^ | 94.2 | ^[12]^ |
| Ni_3_Fe-CO_3_  LDH/Cu | 1 M KOH +  5 mM KNO_3_ | -0.2 | 1.261 mg h^-1^ cm^-2^ | 96.8 | ^[13]^ |
| Cu_11_Ag_3_ | 1 M KOH +  14.3 mM KNO_3_ | -0.33 | 4.573 mg h^-1^ cm^-2^ | 98.5 | ^[14]^ |
| Cu-Co_3_O_4_ | 0.1 M Na_2_SO_4_ +  8.06 mM NaNO_3_ | -0.6 | 0.62 mg h^-1^ mg_cat_^-1^ | 86.5 | ^[15]^ |

**Reference**

[1] G. Kresse, D. Joubert, *Physical Review B* **1999**, *59*, 1758-1775.

[2] J. Heyd, G. E. Scuseria, M. Ernzerhof, *Journal of Chemical Physics* **2003**, *118*, 8207-8215.

[3] J. P. Perdew, K. Burke, M. Ernzerhof, *Phys. Rev. Lett.* **1996**, *77*, 3865-3868.

[4] T. T. Li, Y. F. Yu, M. Y. Pei, *J. Phys. Chem. C* **2023**, *127*, 6271-6279.

[5] S. Grimme, J. Antony, S. Ehrlich, H. Krieg, *Journal of Chemical Physics* **2010**, *132*, 19.

[6] Y. Wang, Y. Xu, C. Cheng, B. Zhang, B. Zhang, Y. Yu, *Angew. Chem.-Int. Edit.* **2024**, *63*.

[7] W. Wang, J. Chen, E. C. M. Tse, *J. Am. Chem. Soc.* **2023**, *145*, 26678-26687.

[8] G.-F. Chen, Y. Yuan, H. Jiang, S.-Y. Ren, L.-X. Ding, L. Ma, T. Wu, J. Lu, H. Wang, *Nature Energy* **2020**, *5*, 605-613.

[9] Y. Wang, W. Zhou, R. Jia, Y. Yu, B. Zhang, *Angew. Chem.-Int. Edit.* **2020**, *59*, 5350-5354.

[10] Y. Wang, H. Li, W. Zhou, X. Zhang, B. Zhang, Y. Yu, *Angew. Chem.-Int. Edit.* **2022**, *61*.

[11] B. Zhou, L. Yu, W. Zhang, X. Liu, H. Zhang, J. Cheng, Z. Chen, M. Li, Y. Shi, F. Jia, Y. Huang, L. Zhang, Z. Ai, *Angew. Chem.-Int. Edit.* **2024**, *63*.

[12] W. He, J. Zhang, S. Dieckhoefer, S. Varhade, A. C. Brix, A. Lielpetere, S. Seisel, J. R. C. Junqueira, W. Schuhmann, *Nat. Commun.* **2022**, *13*.

[13] K.-H. Kim, H. Lee, X. Huang, J. H. Choi, C. Chen, J. K. Kang, D. O'Hare, *Energy Environ. Sci.* **2023**, *16*, 663-672.

[14] C. Wang, Z. Liu, Q. Peng, D. Xing, T. Hu, F. Du, C. Li, R. Ma, H. Yang, C. Guo, *Angewandte Chemie (International ed. in English)* **2024**, e202415259-e202415259.

[15] Z. Niu, S. Fan, X. Li, Z. Liu, J. Wang, J. Duan, M. O. Tade, S. Liu, *Acs Applied Materials & Interfaces* **2022**, *14*, 35477-35484.
